# EGFR/Met association regulates EGFR TKI resistance in breast cancer

**DOI:** 10.1186/1750-2187-5-8

**Published:** 2010-07-12

**Authors:** Kelly L Mueller, Zeng-Quan Yang, Ramsi Haddad, Stephen P Ethier, Julie L Boerner

**Affiliations:** 1 Karmanos Cancer Institute at Wayne State University, John R. St, Detroit, MI 48201, USA; 2 Department of Pharmacology at Wayne State University, E. Canfield, Detroit, MI 48201, USA; 3 Department of Pathology at Wayne State University, Detroit, MI, 48201, USA

## Abstract

Breast cancers show a lack of response to epidermal growth factor receptor (EGFR) tyrosine kinase inhibitors (TKIs), despite 30% of tumors expressing EGFR. The mechanism of this resistance is unknown; however, we have recently shown that Met kinase activity compensates for loss of EGFR kinase activity in cell culture models. Met has been implicated in the pathogenesis of breast tumors and therefore may cooperate with EGFR for tumor growth. Here we have found that EGFR phosphorylation and cell proliferation is in part regulated by Met expression. In addition, we found that Met constitutive phosphorylation occurred independent of the Met ligand hepatocyte growth factor (HGF). Ligand-independent Met phosphorylation is mediated by Met amplification, mutation, or overexpression and by Met interaction with other cell surface molecules. In SUM229 breast cancer cells, we found that Met was not amplified or mutated, however it was overexpressed. Met overexpression did not directly correlate with ligand-independent Met phosphorylation as the SUM229 cell line was the only Met expressing breast cancer line with constitutive Met phosphorylation. Interestingly, Met expression did correlate with EGFR expression and we identified an EGFR/Met complex via co-immunoprecipitation. However, we only observed Met constitutive phosphorylation when c-Src also was part of this complex. Ligand-independent phosphorylation of Met was decreased by down regulating EGFR expression or by inhibiting c-Src kinase activity. Lastly, inhibiting EGFR and Met kinase activities resulted in a synergistic decrease in cell proliferation, supporting the idea that EGFR and Met functionally, as well as physically interact in breast cancer cells to regulate response to EGFR inhibitors.

## Introduction

Epidermal growth factor receptor (EGFR) is a tyrosine kinase receptor shown to be mechanistically involved in cell growth and survival (reviewed in [1]). Ligand activation of EGFR results in homo- and hetero-dimerization with other members of the EGFR family of receptor (reviewed in [1]). This dimerization enables EGFR to autophosphorylate, resulting in the recruitment of signaling proteins to the receptor (reviewed in [1]). Approximately 30% of human breast tumors overexpress EGFR, and this overexpression correlates with a loss of estrogen responsiveness and a poor prognosis [2-5]. Despite strong correlative evidence from human breast tumors, transgenic mouse models have clearly demonstrated that overexpression of the EGFR alone is insufficient for tumor formation [6]. EGFR tyrosine kinase inhibitors (TKIs) are in clinical use in lung and pancreatic cancers, but have yet to demonstrate efficacy in breast cancer. We and others have recently identified the receptor tyrosine kinase Met as a key regulator of EGFR tyrosine kinase inhibitor resistance in cancer [7,8].

Met also is overexpressed in breast cancer cells and human breast tumors and its expression correlates with EGFR expression in basal type breast cancers [9-11]. Met or hepatocyte growth factor receptor is characterized as a receptor tyrosine kinase [12]. However, unlike EGFR, there are two broad mechanisms of Met activation: ligand-dependent and ligand-independent. In the mammary gland, ligand-dependent activation of Met involves the paracrine production of HGF by stromal cells, including fibroblasts [13]. Ligand-independent activation of Met has been shown to occur through a number of mechanisms, including mutation of Met, constitutive dimerization of Met associated with overexpression, pathway activation via hypoxic conditions, transactivation by other membrane proteins (including EGFR), and loss of negative regulators [14].

We have previously shown that the kinase activity of Met, in part, regulates EGFR tyrosine phosphorylation and growth in the absence of EGFR tyrosine kinase activity. Here we have identified a physical and functional interaction between EGFR and Met. Specifically, we found that EGFR tyrosine phosphorylation and growth were in part dependent on the expression of Met. We also found that neither HGF mRNA nor protein was expressed, suggesting a ligand-independent mechanism of Met phosphorylation. In that regard, Met was not amplified or mutated in SUM229 cells. The protein expression of Met was increased in the SUM229 cells, yet an increase in protein expression did not correlate with ligand-independent Met phosphorylation. Instead, we found that EGFR and Met co-immunoprecipitated in the SUM229 cells both in the absence and in presence of gefitinib and down regulation of EGFR expression decreased Met constitutive phosphorylation, again supporting a physical and functional interaction between EGFR and Met. Interestingly, c-Src was part of the EGFR/Met complex when Met was constitutively phosphorylated and inhibiting c-Src kinase activity also decreased Met phosphorylation. Taken together, these data suggest that EGFR and Met interact both physically and functionally and that the interaction is independent of the kinase activities of both molecules and that this interaction promotes EGFR TKI resistance via constitutive phosphorylation of Met.

## Results

### Met expression regulates EGFR tyrosine phosphorylation and growth in the presence of gefitinib

**Figure 1:**
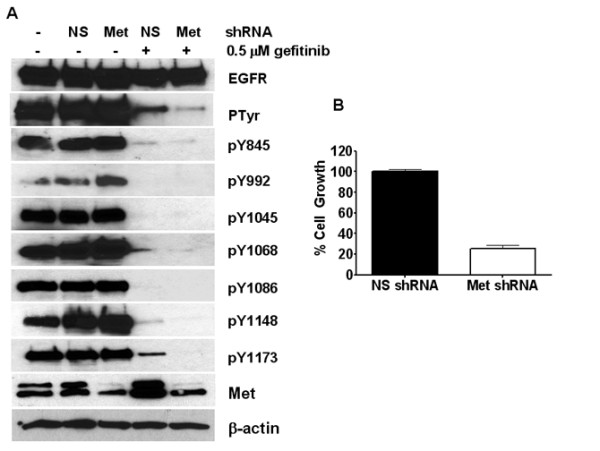
**Met expression mediates EGFR tyrosine phosphorylation and proliferation in the absence of EGFR kinase activity**. (A) SUM229 cells were infected with lentiviral particles containing Met shRNA or a non-silencing shRNA for 72 hrs. Cells were treated with gefitinib for the last hour of infection at 0.5 μM, cells were lysed, and lysates were immunoprecipitated with anti-EGFR. Immunoprecipitates were immunoblotted with anti-EGFR, anti-Ptyr, and the indicated phospho-specific sites on the EGFR. Corresponding whole cell lysates were immunoblotted with anti-Met and β-actin as a loaded control. Through quantification, EGFR phosphorylation was decreased by 53% in the gefitinib treated cells and 88% in the gefitinib with Met knocked down cells. The two bands in the Met immunoblot represent processed and pro-forms of Met. (B) SUM229 cells were infected with lentiviral particles containing Met shRNA in the presence of gefitinib or a non-silencing control for seven days in the presence of puromycin to select for infected cells. Cells were counted using a Coulter Counter and the day 8 values were graphed with the error bars representing SEM.

We have previously shown that constitutive phosphorylation of Met contributes to EGFR TKI resistance in breast cancer and that decreasing Met kinase activity, decreased EGFR tyrosine phosphorylation and proliferation in the presence of an EGFR tyrosine kinase inhibitor [7]. However, to determine if the expression of Met is required for EGFR tyrosine phosphorylation and growth in the presence of EGFR TKIs, we used lentiviral encoding shRNA constructs targeting Met. Using this method, we were able knockdown Met to approximately 40% of its endogenous expression level (Fig. 1A; Met panel). When these cells containing the knocked down Met were analyzed for EGFR tyrosine phosphorylation in the presence of EGFR TKIs, we observed a decreased in EGFR tyrosine phosphorylation (Fig. 1A; PTyr panel). This decrease in EGFR overall tyrosine phosphorylation appeared to be mediated by a decrease in the phosphorylation of tyrosines 1068, 1148, and 1173 (Fig. 1A). Importantly, when the Met knockdown cells were analyzed for cell proliferation in the presence of gefitinib, an 80% decrease in cell proliferation was observed (Fig. 1B). These data suggest that downregulating Met expression sensitizes cells to EGFR TKIs by decreasing EGFR tyrosine phosphorylation and proliferation.

### Met is not genomically amplified but is overexpressed in EGFR expressing breast cancer cells

**Figure 2:**
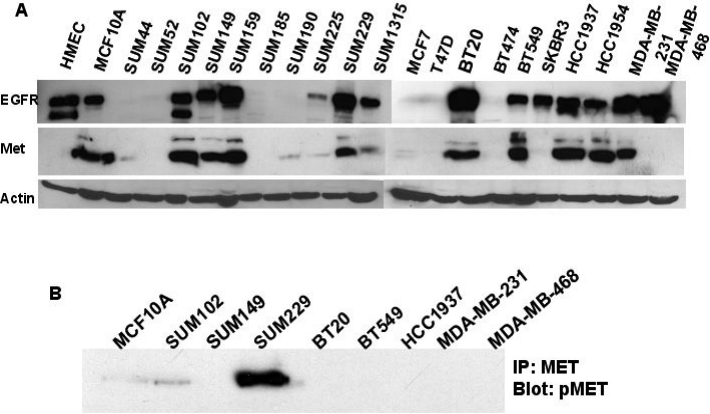
**Met is not amplified, but is overexpressed at the protein level**. (A) Lysates from the indicated breast cancer cell lines was prepared and 100 ug of protein was separated by SDS-PAGE, transferred, and immunoblotted with anti-EGFR, anti-Met, and anti-β-actin. (B) Lysates from the Met expressing breast cancer cell lines were immunoprecipitated with anti-Met antibodies. Immunoprecipitates were separated by SDS-PAGE, transferred, and immunoblotted with anti-pMet antibodies.

The mechanism of Met constitutive phosphorylation in SUM229 breast cancer cells is unknown. It was previously reported that under conditions of acquired resistance to gefitinib in a lung cancer cell line, Met becomes genomically amplified and overexpressed [8]. Therefore, we analyzed array comparative genomic hybridization data to determine if Met was amplified in the SUM229 cells [15,16]. We found that Met was not amplified in SUM229 cells as determined by statistical analysis using circular binary segmentation, which indicated that, the copy number of MET was not more than 1.3 fold (data not shown). In addition, we sequenced the Met juxtamembrane and kinase domain regions previously shown to be mutated and mediate constitutive activation of Met in renal cancers [17]. No mutations were detected in Met from cDNA prepared from SUM229 cells (data not shown). Lastly, to determine if Met protein expression was increased in SUM229 cells we compared protein lysates from SUM229 cells with lysates from a panel of twenty breast cancer cell lines and two non-malignant mammary epithelial cell lines (Fig. 2A). From this analysis, we found that Met protein was expressed in 50% of the breast cancer cell lines, yet only the SUM229 cell line contained constitutively phosphorylated Met (Fig. 2B).

### Met constitutive phosphorylation occurs independent of ligand in EGFR TKI resistant breast cancer cells

**Figure 3:**
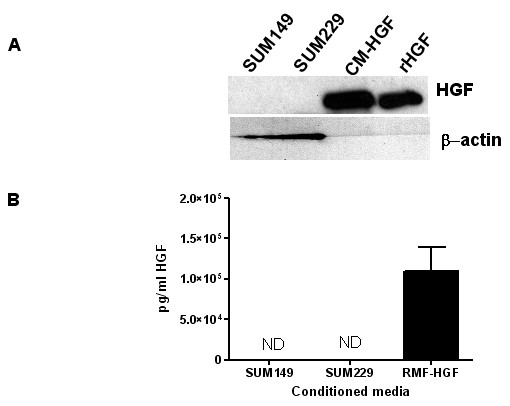
**HGF is not expressed in SUM229 breast cancer cells**. (A) Lysates from SUM149 and SUM229 and conditioned media from RMF-HGF cells were immunoblotted with anti-HGFα. Recombinant HGF was used as a positive control. (B) Conditioned media was collected from confluent SUM149, SUM229, and RMF-HGF cell cultures. Diluted conditioned media was analyzed for HGF expression using ELISA with RMF-HGF as a positive control. The amount of HGF produced from the SUM149 and SUM229 lysates was undetectable.

**Figure 4:**
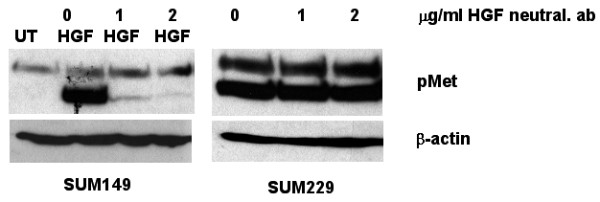
**Met phosphorylation occurs independent of ligand**. SUM149 and SUM229 cells were treated with increasing concentrations of anti-HGF neutralizing antibody. The SUM149 cells were stimulated with 50 ng/ml HGF to induce Met phosphorylation. Whole cell lysates were immunoblotted with anti-phospho-Met.

To determine if constitutive phosphorylation of Met is mediated by its ligand, HGF, we first determined the amount of HGF present in SUM229 cells from both cell lysates and conditioned media. We were unable to detect HGF expression either in the cell lysate (Fig. 3A) or in conditioned media (Fig. 3B). Lysates and conditioned media from cells engineered to overexpress HGF were used as positive controls (Figs. 3A and 3B). These results were not unexpected, as HGF is not normally produced as an autocrine factor in normal epithelial nor in carcinomas [18]. However, to eliminate the possibility of a small amount of HGF contributing to the constitutive phosphorylation of Met, we used an HGF neutralizing antibody to prevent HGF from binding to Met. In the SUM149 cells where Met is not constitutively phosphorylated, we added exogenous HGF to stimulate Met phosphorylation and found that the neutralizing antibody decreased HGF-mediated Met tyrosine phosphorylation (Fig. 4; left panel). Yet in the SUM229 cells where Met is constitutively phosphorylated, the HGF neutralizing antibody had no effect on Met phosphorylation (Fig. 4; right panel). Therefore, Met is activated by a ligand-independent mechanism in SUM229 cells.

### EGFR associates with Met in EGFR TKI resistant breast cancer cells

**Figure 5:**
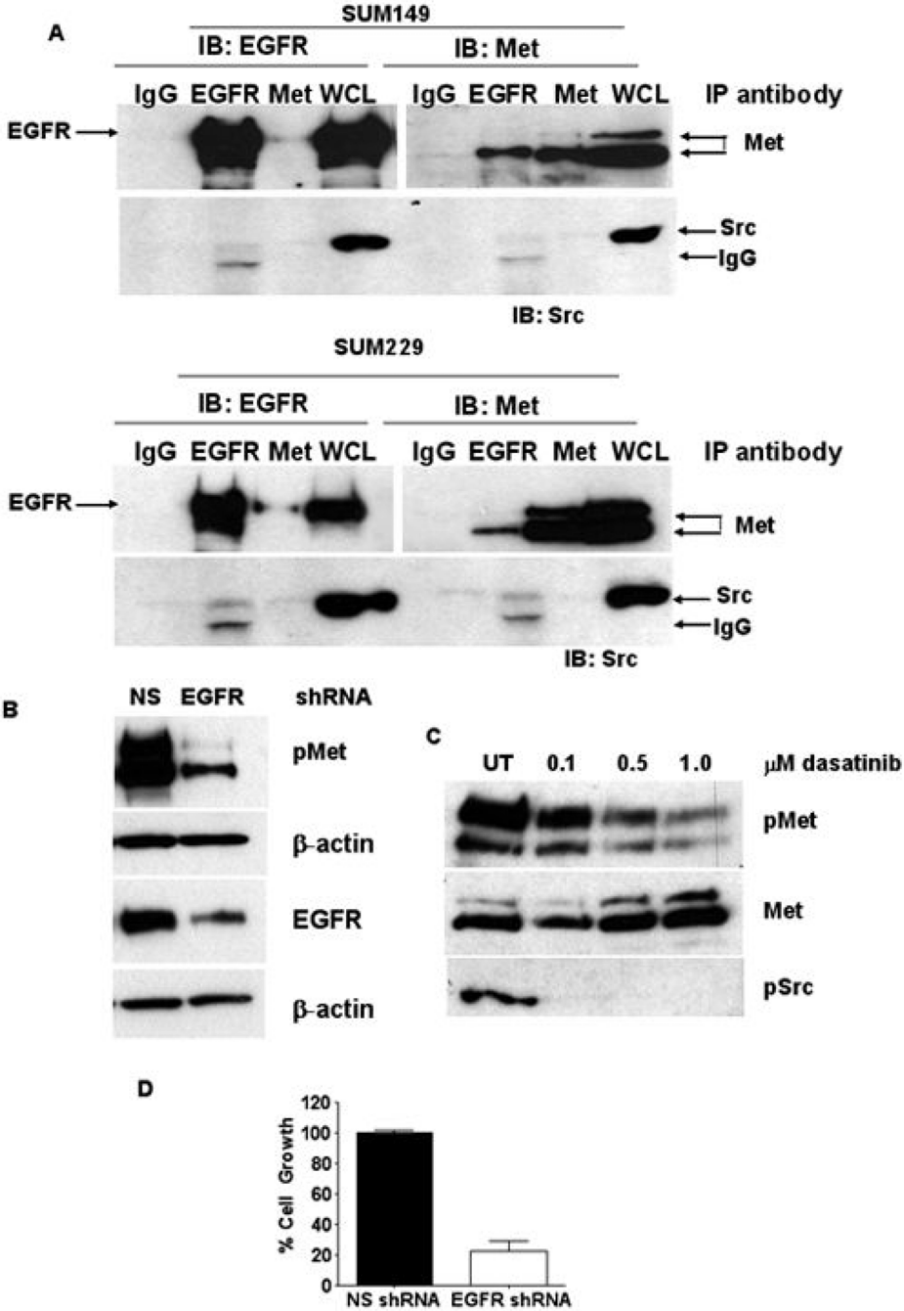
**EGFR and Met association independent of EGFR kinase activity**. (A) SUM149 or SUM229 cells were treated with 0.5 μM gefitinib or DMSO control for 30 min. Cells were lysed and immunoprecipitated with anti-EGFR, anti-Met, and anti-IgG. Whole cell lysates were used as a control. Immunoprecipitates were separated by SDS-PAGE, transferred, and immunoblotted using anti-EGFR, anti-Met, or anti-c-Src antibodies. (B) SUM229 cells were incubated with EGFR shRNA lentiviral particles for 72 hrs. Lysates were prepared and separated by SDS-PAGE. Membranes were immunoblotted with anti-pMet, anti-Met, anti-EGFR, and β-actin. (C) SUM229 cells were treated with increasing concentrations of dasatinib for 2 hrs. Lysates were prepared and immunoblotted with anti-pMet, anti-Met, and anti-pSrc antibodies. (D) SUM229 cells were incubated with EGFR shRNA lentiviral particles for 24 hrs and then treated with puromycin to select for virus expressing cells. After the puromycin was added for 24 hrs, the cells were grown for 7 days at which point cell numbers were determined via Coulter counting.

Ligand-independent activation of Met has been shown to occur through association with co-receptors. These co-receptors include molecules involved in adhesion, such as integrins and CD44, receptors known as semiphorins or plexins, and receptor tyrosine kinases, such as EGFR and Ron [19]. Therefore, to determine if EGFR and Met have the ability to co-associate, we immunoprecipitated lysates from SUM149 or SUM229 cells using EGFR or Met antibodies (as well as an isotype antibody control) and immunoblotted using EGFR or Met antibodies. We found that EGFR and Met did indeed co-immunoprecipitate both in SUM149 and SUM229 cells (Fig. 5A). Our previous work found that Met contributed to EGFR tyrosine phosphorylation in the presence of an EGFR TKI [7]. We hypothesized that this was a direct phosphorylation that occurred via EGFR and Met association. To validate this hypothesis we treated SUM149 and SUM229 cells with gefitinib and repeated the co-immunoprecipitation experiment. As predicted, we found that EGFR and Met remained associated independent of EGFR tyrosine kinase activity (data not shown). These results suggest that Met may be mediating EGFR phosphorylation through co-association leading to conformation changes in both receptors and that the association between EGFR and Met may regulate the kinase activity of Met. However, because this association is observed in cells without ligand-independent Met phosphorylation we looked for another kinase associating with the EGFR/Met complex in SUM229 cells. We identified c-Src associating with the EGFR in the immunoprecipitation complex in the SUM229 cells, but not the gefitinib sensitive SUM149 cells (Fig. 5A). These data complement our previous studies demonstrating a role for c-Src in phosphorylating EGFR in the absence of EGFR kinase activity [7].

To determine if the formation of the co-precipitation complex was regulating ligand-independent Met phosphorylation, we used shRNA to downregulate EGFR expression and found that decreasing EGFR expression, thereby decreasing EGFR and Met association, reduced Met phosphorylation (Fig. 5B). In addition, inhibiting c-Src kinase activity with the small molecule kinase inhibitor dasatinib also reduced Met phosphorylation (Fig. 5C). Therefore, it can be concluded that breaking apart the EGFR/Met association and reducing the contribution of c-Src kinase activity by decreasing EGFR expression, diminished Met constitutive phosphorylation. Thus, decreasing EGFR expression should abrogate the growth of SUM229 cells. In fact, knocking down EGFR expression decreased cell proliferation over a 7-day period by 80% (Fig. 5D). These data suggest that EGFR has the ability to act as a scaffolding protein to link Met and c-Src.

### **EGFR and Met inhibitors act synergistically to abrogate EGFR TKI resistant breast cancer cell growth**

**Figure 6:**
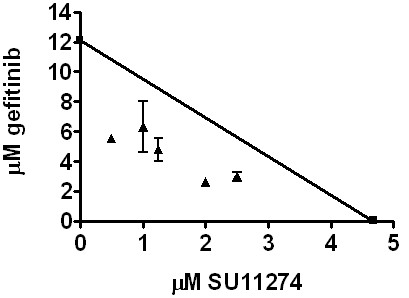
**EGFR and Met inhibitor synergy in SUM229 cells**. SUM229 cells were treated with various concentrations of gefitinib and SU11274 and the IC_50 _of gefitinib was calculated for each concentration of SU11274. A line was drawn between the IC_50 _for gefitinib (y-axis; 12.2 μM) and SU11274 (x-axis; 4.8 μM). The calculated IC_50 _values for gefitinib with SU11274 combination treatment were plotted. Points falling below the line represent synergistic drug interactions.

The association of EGFR and Met and their mutual regulation of each other in the absence of EGFR kinase activity and Met ligand suggests that inhibiting the kinase activity of the molecules individually may be insufficient to abrogate cell growth. We previously published proliferation assays with the combination of EGFR and Met inhibitors and found when SUM229 cells were treated with this combination of inhibitors, cell proliferation was arrested [7]. However, we did not determine if this decrease in cell proliferation was a result of synergism between the two drugs. Therefore, we used cell viability assays to assess the IC_50 _values for gefitinib and SU11274 (a Met inhibitor) and performed synergy analysis using an isobologram. We found the calculated IC_50 _for gefitinib to be approximately 12.2 μM and the IC_50 _for SU11274 to be 4.8 μM (Figure 6). Adding increasing concentrations of SU11274 to gefitinib treated cells reduced the IC_50 _of the cells to geftinib. CI values were calculated and values under 1 are considered synergistic. When the SUM229 cells were treated with gefitinib and SU11274, the calculated CI values were less than 1. Specifically, at 1.25 μM SU11274 the mean CI value was 0.619 ± 0.038 and at 2.5 μM SU11274 the CI value was 0.781 ± 0.025. Therefore, EGFR and Met kinase inhibitors interact synergistically in the SUM229 cells.

## Discussion

We have shown that in an EGFR TKI resistant breast cancer cell line, Met was constitutively activated independent of ligand. Specifically, HGF was not produced by these cells and an inhibitory antibody against HGF did not block Met phosphorylation. This ligand-independent activation of Met occurred in part by increased Met expression and in part by association with EGFR. This association was independent of EGFR kinase activity. Breaking apart this association by downregulating EGFR expression dramatically decreased Met phosphorylation and inhibited cell growth. EGFR also co-precipitated with c-Src and inhibiting c-Src kinase activity decreased Met phosphorylation. In addition, inhibiting both EGFR and Met kinase activities decreased cell viability synergistically. Therefore, these data demonstrate that an association between EGFR, Met, and c-Src regulates Met constitutive phosphorylation and growth in SUM229 breast cancer cells.

In the mammary gland, HGF is produced in stromal tissue, including mammary fibroblasts, but not in the epithelial cells [20,21]. Therefore, in the mammary gland, Met is activated by HGF via a paracrine mechanism. With respect to breast carcinomas, the localization of HGF within the mammary gland is less clear. Yamashita and colleagues found that in both human breast tumors and two breast cancer cell lines, HGF was not expressed or secreted [22]. In contrast, several groups have suggested that HGF is expressed in the breast tumor cells via mRNA detection using *in situ *hybridization [23,24]. In this study, we demonstrate that HGF protein is not expressed or secreted in two breast cancer cell lines, SUM149 and SUM229. These data support the notion that HGF is primarily produced in stromal tissue in breast cancers and therefore studying the role of HGF/Met signaling using cell culture models may overlook important contributions of the tumor microenvironment to EGFR/Met crosstalk in breast cancer. In that regard, we have previously shown that the SUM149 cells responded to exogenous HGF treatment by phosphorylating Met and recovering some EGFR tyrosine phosphorylation and growth in the presence of EGFR TKIs [7].

Our data support a ligand-independent mechanism of Met activation in SUM229 breast cancer cells. Lai and colleagues summarized multiple mechanisms for ligand-independent Met activation [14]. Specifically, Met amplification, mutation, and truncation, overexpression of Met leading to constitutive dimerization, pathway activation by hypoxia, transactivation by other receptors, and loss of negative regulators have been shown to induce ligand-independent Met activation [14]. Our data clearly demonstrate that Met is not amplified, mutated, or truncated in SUM229 cells. However, we do find that Met is overexpressed at the protein level and that EGFR associates with Met. We have therefore provided evidence for the interaction and dependence of Met phosphorylation by EGFR expression and kinase activation.

Both physical and functional associations of EGFR and Met have been described previously. Specifically, EGFR and c-Met co-associate in normal human hepatocytes and lung cancer cell lines [25,26]. In a wider array of models, EGFR and Met have been shown to crosstalk functionally. EGFR has been shown to activate Met in lung and thyroid cancer cells as well as in the development of the kidney, hepatocytes, and retina [27-32]. This is the first report of EGFR and Met physically interacting and EGFR regulating Met activation in the absence of HGF in breast cancer.

Cellular mechanisms of resistance to EGFR small molecule inhibitors have best been studied in lung cancers containing EGFR sensitizing mutations. Using clinical specimens from patients with non small cell lung cancers that recurred after treatment with EGFR inhibitors, 50% of patients have acquired an additional mutation, T790 M, which creates an EGFR that no longer responds to EGFR inhibition [33]. In addition, 20% of EGFR inhibitor refractory lung cancers have amplification of Met [8,34]. Other molecules such as Ras and IGF-IR also have been implicated in resistance to EGFR inhibitors [35,36]. The data provided in this report suggest that in addition to the identified resistance factors in lung cancer, Met activity may be a regulator of response to EGFR inhibitors in breast cancers.

Overall, our data provide evidence for a physical and functional interaction between EGFR and Met. This interaction regulates the phosphorylation of both EGFR and Met such that each molecule is capable of mediating cellular responses independent of EGFR kinase activity. In addition, this EGFR kinase independent activity is in part mediated by c-Src activity. Therefore, the abrogation of the kinase activity of each molecule in combination provides intriguing evidence for dual EGFR and Met inhibitor studies in breast cancers.

## Materials and methods

### Cell lines, culture conditions, and reagents

The growth conditions for each cell line are as follows. SUM 52, SUM 149, SUM 159, SUM 185, SUM 225, and SUM 229 cells are grown in 5%IH media (Ham's F-12 media, supplemented with 5% FBS, 1 μg/ml hydrocortisone, and 5 μg/ml insulin). SUM 1315 cells are grown in 5%IE media (Ham's F-12 media, supplemented with 5% FBS, 10 ng/ml EGF, and 5 μg/ml insulin). SUM 44 and SUM 190 cells are grown in SFIH media (Ham's F-12 media, supplemented with 1 μg/ml hydrocortisone, 5 μg/ml insulin, 5 mM ethanolamine, 10 mM HEPES, 5 μg/ml transferrin, 10 nM triiodo-thyronine, 50 μM sodium selenite, and 5% BSA). SUM 102 and MCF10A cells are grown in SFIHE media (Ham's F-12 media, supplemented with 1 μg/ml hydrocortisone, 5 μg/ml insulin, 10 ng/ml EGF, 5 mM ethanolamine, 10 mM HEPES, 5 μg/ml transferrin, 10 nM triiodo-thyronine, 50 μM sodium selenite, and 5% BSA). MCF7, SKBr3, T47 D, MDA-MB-231 and MDA-MB-468 cells are grown in DMEM+10%FBS media (DMEM media, supplemented with 10% FBS). BT-20 cells are grown in Eagles+NEAA media (Eagle's MEM with 2 mM L-glutamine and Earle's BSS adjusted to contain 1.5 g/L sodium bicarbonate, 0.1 mM non-essential amino acids, 1 mM sodium pyruvate, and 10% FBS). BT-549 cells are grown in RPMI+L-GLUT(2 mM) media (RPMI-1640, supplemented to contain 1.5 g/L sodium bicarbonate, 4.5 g/L glucose, 10 mM HEPES, 1 mM sodium pyruvate, 0.023 IU/ml insulin, and 10% FBS). HCC 1937 and HCC 1954 cells are grown in RPMI+L-GLUT media (RPMI-1640 media with 2 mM L-glutamine adjusted to contain 1.5 g/L sodium bicarbonate, 4.5 g/L glucose, 10 mM HEPES, 1 mM sodium pyruvate, and 10% FBS). The SUM and HCC cells are cultured in 10% CO_2 _and the remaining cells are cultured in 5% CO_2_. All media are supplemented with 2.5 μg/ml amphotericin B and 25 μg/ml genatimicin.

Gefitinib was provided by AstraZeneca, SU11274 was purchased from EMD Biosciences (Gibbstown, NJ), and dasatinib was purchased by LC Laboratories (Wouburn, MA). The HGF neutralizing antibody was a kind gift from George Vande Woude (VARI, Grand Rapids, MI). All other reagents were purchased from Thermo Fisher (Houston, TX) or Sigma (St. Louis, MO), unless indicated.

### Genetic analysis

The genomic array CGH experiments were performed previously using the Agilent 44 K human genome CGH microarray chip [37,38](Agilent Technologies, Palo Alto, CA, USA). Circular binary segmentation analysis was used to determine changes in copy number [39].

### HGF ELISA

One million cells were plated on 100 mm dishes and grown for 48 hours. The media was changed to serum free media and the cells were incubated for 72 hours. Conditioned media was collected from fibroblasts engineered to express HGF as a positive control. The HGF ELSA assay was performed as directed by the manufacture (R&D Systems, Minneapolis, MN). Briefly, conditioned media was undiluted or diluted 1:2 and 1:10 and used to measure the amount of HGF using a standard curve.

### Immunoprecipitation and Immunoblotting

Cells were plated at 1 million cells per 100 mm dish and growth for 48 hours. Cells were then lysed in CHAPs lysis buffer (10 mM CHAPs, 50 mM Tris, pH 8.0, 150 mM NaCl, and 2 mM EDTA with 10 μM NaOVa and 1× protease inhibitor cocktail (EMD Biosciences)). For immunoprecipitations 500 μg of lysate was precleared with 40 μl of protein A agarose beads (Millipore, Billerica, MA) for 30 min at 4°C. The supernatant was saved and added to 5 μl of mab-108 EGFR antibody (kind gift from Michael Weber, University of Virginia), Met antibody (Cell Signaling, Beverly, MA), or IgG isotype control antibody (Millipore) for 1 hr at 4°C. Immunoprecipitates were collected with the addition of 40 μl of protein A agarose beads for 30 min at 4°C and washed 2× with CHAPs and 1× with PBS. The protein was removed from the beads with 40 μl of hot 2× lameli buffer. Protein was separated by SDS-PAGE, transferred to Immunolin-P (Millipore) and immunoblotted for the indicated protein.

For immunoblotting, indicated amount of protein lysate was separated by SDS-PAGE and transferred to Immobolin-P. Membranes were blocked in either 5% non-fat dry milk or 5% BSA for 1 hr at RT. The following primary antibodies were used in the experiments: anti-EGFR (Cell Signaling, 1:500), anti-Met (Cell Signaling 1:500), anti-PTyr-HRP (Invitrogen, Carlsbad, CA, 1:5 000), anti-EGFR Y845 (Cell Signaling, 1:750), anti-EGFR Y992 (Cell Signaling, 1:500), anti-EGFR Y1045 (Cell Signaling, 1:500), anti-EGFR Y1068 (Cell Signaling, 1:500), anti-EGFR Y1086 (Cell Signaling, 1:500), anti-EGFR Y1148 (Cell Signaling, 1:500), anti-EGFR Y1173 (Cell Signaling, 1:750), anti-pMet (Cell Signaling, 1:500), anti-HGF (IBL, 1:500), anti-Src (Cell Signaling,1:1 000), and anti-β-actin (Sigma, 1:10 000). The antibodies were incubated overnight at 4°C, with the exception of the PTyr antibody which was blocked overnight and incubated with antibody for 1 hr. The membranes are then washed with TBS-T (TBS + 0.1% Tween-2) three times for 10 min each followed by incubation with the corresponding secondary antibody, and another series of three washes. Incubation with enhanced chemiluminescence (GE Biosciences, Piscataway, NJ) followed by exposure to film was used to detect the reactive bands. Each experiment was repeated at least three times and quantitated using densitometry.

### Lentiviral shRNA knockdown

To downregulate EGFR and Met expression we used shRNA lentiviral particles using commercially available lentiviral constructs from OpenBiosystems (Huntsville, AL)(EGFR = TRCN0000121204 and Met = TRCN0000121233). Twenty-four EGFR shRNA constructs and twelve Met shRNA constructs were screened and validated for EGFR or Met knockdown. At least three constructs were used in the studies for each protein with the data from the representative shRNA shown. The lentiviruses were packaged using a third generation lentiviral packaging system developed by Didier Trono and colleagues (Lausanne, Switzerland) and purchased from Addgene [40]. Specifically, Addgene plasmids pMLDg/pRRE (12251), pRSV-Rev (12253), and pMD2.G (12259) were transfected into HEK293T cells with the lentiviral vectors containing the shRNAs using FUGENE6 (Roche, Madison, WI). Cellular supernatant was collected on days 2 and 3 after transfection, pooled, and filtered. The lentivirus was titered using HEK293T cells incubated with increasing concentrations of virus with polybrene and selected for via the puromycin selection on the lentiviral vector. Colonies were counted and used to compare viral preps and between viruses for consistent titers used in experiments. For the SUM229 cells, equal amounts of virus was added to SUM229 cells in the presence of polybrene for four days prior to cell lysis.

### Cell proliferation assays

For the proliferation assays the indicated breast cancer cells were plated in triplicate in 6-well plates at 35 000 cells per well (Day 0). The next day, the cells were treated with gefitinib every day for seven days at the indicated dosage. The number of cells was determined using a Coulter Counter on Days 1, 4, and 8. Each experiment was repeated at least twice and the graph represents the average and standard error of the mean at day 8.

For the MTS assays, cells were plated at 2 000 cells/well of a 96 well plate in triplicate. The indicated doses of gefitinib and SU11274 were added 24 hours later. The cells were incubated with the drugs for 72 hours at which time the MTS reagent was added per manufacture directions (Promega, Madison, WI) and read using a Dynex spectrophotometer. The experiment was repeated three times with error bars representing the SEM.

### Statistics

Isobolograms were performed by determining the IC_50 _values for gefitinib and SU11274 using Graph Pad Prism via standard MTS assay. The concentration of gefitinib used included 0.001, 0.1, 1, 5, 10, 50, and 100 μM. The concentrations of SU11274 used included 0.01, 0.1, 0.5, 1, and 5 μM. CI values were calculated using the following equation: (IC_50 _combination/IC_50 _gefitinib) + (concentration of SU11274/IC_50 _SU11274). CI values were compared to 1.0 (CI value with 0 μM SU11274) by unpaired T tests.

## Conflict of Interest

The authors declare that they have no competing interests.

## Authors' contributions

KLM performed the experiments for figures 1, 3, 4, 5, 6. Z-QY generated performed the array CGH and microarray data used to determine that Met was not amplified or overexpressed. RH analyzed the data generated by Zeng-Quan Yang to determine statistical significance. SPE contributed significant intellectual support to this manuscript as well as supports RH. JLB performed the experiments for figure 2 and assistant with some of the other experiments. She also wrote the manuscript and directed the project. All authors read and approved this manuscript.
